# Self-perceived health and quality of life among Azorean deportees: a cross sectional descriptive study

**DOI:** 10.1186/s40064-016-3219-9

**Published:** 2016-09-15

**Authors:** Maryellen D. Brisbois, Helena Oliveira Silva, Helder Rocha Pereira, Kristen A. Sethares

**Affiliations:** 1 University of Massachusetts Dartmouth, 285 Old Westport Road, Dartmouth, MA 02747 USA; 2 Escola Superior de Saúde da Universidade dos Açores Secção de Ponta Delgada, Apartado 1422, 9501–855 Ponta Delgada, Ladeira da Mãe de Deus, Azores Portugal

**Keywords:** Deportation, Physical health, Mental health, Quality of life, Self-report

## Abstract

**Background:**

Immigration policies can cause significant public health consequences, posing detrimental social and health effects for migrants, their families and communities. Migrants often face obstacles to health due to access, discrimination, language and cultural barriers, legal status, economic difficulties, social isolation, and fear of deportation. The process of deportation has become more rapid and frequent in the U.S. with inadequate health information in the literature regarding this relocated population post-deportation. The PROMIS^®^ Global Health Short Form was used to measure the self-reported QOL, physical and mental health of male deportees from the US to Portugal from 2009 to 2013.

**Findings:**

Twenty five males aged 28–64 years who had been deported from the US to Portugal participated in the study. Overall, their EuroQol, Global Physical Health and Global Mental Health Scores were below the established tool mean, with self-reported mental health having the lowest score. Age, marital status, length of time in the US prior to deportation, and length of time since deportation may impact the well-being of deportees post deportation.

**Conclusions:**

Study results suggest the deportees in this study were less healthy than the general population. Future research and tailored initiatives regarding the overall health of deportees, with a focus on quality of life and mental health should be conducted to better understand their impact on reintegration. Overall study scores were lower than mean tool scores indicating the need for more research in this vulnerable group to support clinical practice and health policy to improve their overall QOL and health through intervention work.

## Background

Individuals have migrated in search of new opportunities and a better life for themselves and their families since the beginning of civilization (International Organization for Migration 2013). These mobile populations often encounter human rights repercussions and health disparities, both for themselves and their families left behind (Fleishman et al. 2015; World Health Organization 2015). Migrants can also face obstacles to health due to access, discrimination, language and cultural barriers, legal status, economic and social difficulties, social isolation, and fear of deportation (Fleishman et al. 2015; Hacker et al. 2012); with migration policies having the potential to cause significant public health consequences (World Health Organization 2015; Gushulak and MacPherson 2006). Migration can be voluntary or forced. ‘Forced’ migration or deportation results from a formal administrative or criminal legal proceeding leading to the removal of a person from the country in which they currently live (Rosenblum and Meissner 2014).

Deportation has a protracted history, but has become more frequent over the past several decades (Dolan et al. 2016) due to the dynamic, constantly changing social, political and economic climate (Ayon 2009), with many undocumented young adults arriving in the US as children being unaware of their immigration status (Suarez-Orozco et al. 2011). There is considerable evidence to support that current immigration policies and their enforcement may have detrimental social and chronic health effects for the migrants, their children, families and communities in the US and abroad (Blake 2014; Brabeck et al. 2014; Morris and Palazuelos 2015). However, there is inadequate health information in the literature regarding this relocated population post-deportation (Vallet 2012; Hilfinger Messias et al. 2015).

## Background and significance

### Immigration law in the United States

Over the past two decades, as a result of the Antiterrorism and Effective Death Penalty Act of 1996, and the Illegal Immigration and Immigrant Responsibility Act of 1996 (IIRIRA 96) (Immigration in America 2012), the process of deportation has become more rapid and frequent in the US One of the most controversial items in IIRIRA 96, Title III, addressed the issue of undocumented aliens already inside US borders (Department of Homeland Security 2013). Both ‘Acts’ made deportation from the US mandatory for legal permanent and non-permanent residents ‘sentenced’ for a year or more, or for minor criminal offenses; with a record high of nearly 400,000 individuals deported from the US in both 2010 and 2011 (Department of Homeland Security 2013).

Many of these deportees have resided in the U.S. since early childhood, leaving behind jobs, pensions, homes, partners, children, parents, and other family members who are US citizens or permanent residents (Bernstein 2004; Lonegan 2007). They are then ostracized in a homeland they have barely known, without family, support systems, understanding of culture and language, housing, and employment or economic opportunities (Moniz 2004). The deportation process can begin suddenly and without warning (Lonegan 2007). Many deportees have not had the benefit of legal counsel in their deportation proceedings, and the opportunity to pursue all available avenues of relief (Human Rights Watch 2009). Yet once they have left the country, deportees are generally barred from reopening their removal proceedings, and are frequently barred for life from returning to the US Bernstein (2004).

### Azorean migrants

There are currently over one million Portuguese-American men, women and children residing in the US, with many from the Azores, Portugal (Library of Congress 2010). This group has migrated to the US for the past 200 years and have made “significant, but still little recognized contributions to American society” (Williams 2007). Once settled, they are considered lawful permanent residents; with the US government granting them the privilege of living in the country for an indefinite amount of time, with the opportunity to become citizens (Richardson 1971). While living in the U.S., they are able to attend school, raise families, gain employment, and pay taxes. But unlike citizens, they can be deported at any time if they are convicted of any of a wide variety of crimes (Shaughnessy 1952).

### Deportation from the US to the Azores

There has been a long diplomatic history of migration between Portugal and the US, more specifically with Azorean residents who relocate to south east (SE) Massachusetts. More recently, in response to IIRIRA 96, the US and Portugal have agreed bilaterally to facilitate a protocol that allowed for a smooth transition for deportees. Locally, the Regional Government of the Azores and SE Massachusetts immigration agencies, detention and correctional facilities have partnered to coordinate their relations and efforts to provide support to aid in the deportees transition toward reintegration in their homeland. From 1987 to 2012, the Azores received 1175 deported persons from other countries. During this time, with the exception of three years, more than 70 % of those deportees were from the US (Nunes Rocha and Borralho 2012). The main reasons for deportation from the US to the Azores were related to illegal residency, drugs, crimes (i.e., robbery), domestic violence and sexual violence (Nunes Rocha and Borralho 2012).

### Health consequences of deportation

Health is defined as “a state of complete physical, mental and social well-being and not merely the absence of disease or infirmity” (World Health Organization 1946). From a nursing perspective, health is viewed through a holistic lens that includes physical, mental, emotional and spiritual perspectives, with migrant health a priority. It is noted that Azorean deportees often struggle to reintegrate as a result of limited or no Portuguese or English language abilities (Moniz 2004). Once deported, there may be few family connections, and challenges with cultural and social skills. In addition, they often suffer from chronic illnesses associated with addiction to controlled substances, with co-morbidities that include AIDS and Hepatitis B (Moniz 2004), as well as emotional and traumatic distress post-deportation (Vallet 2012). The literature describes several short and long term consequences of detention and deportation on an individual and family. These include: (1) the trauma of sudden and imposed family separation; (2) financial, health-related, social and psychological consequences for the deportee; (3) changes in family structure causing instability; (4) financial burden for families; and (5) negative impact on the broader community (Brabeck et al. 2014; Morris and Palazuelos 2015). These consequences are considered the ‘collateral damage’ of deportation (Bernstein 2004).

The health and well-being of deportees was identified by the researchers as a health priority through key informants in both the US and Portugal, as little is known about the health status of this vulnerable aggregate. There are few studies in the literature pertaining to nursing research and the effect of deportation of US immigrants, their families and communities. Understanding this perspective is essential to conduct future health intervention research that support reintegration, improving health outcomes and addressing health policy. Therefore, the purpose of this study is to measure the self-reported quality of life, physical and mental health among deportees from the US to Portugal.

### Research trajectory

This study is part of a growing body of research with nursing faculty and students from a university in northeast US and a university in Portugal, with a focus on the impact of deportation on detainees, deportees, and their families. As part of this long-term collaborative effort, data collection on two associated studies is complete, with preliminary findings discussed with the community of interest in Portugal. Key informants in the US also identified the relatives of deportees as being a potentially vulnerable group; therefore, an IRB approved study is underway regarding this phenomenon. And finally, an additional IRB approved study regarding the health of detainees in the US awaiting deportation to Portugal is ongoing.

## Methods

### Design

A cross sectional descriptive study was designed to measure the global quality of life (QOL), physical and mental health of twenty five (25) Azorean male deportees recruited from two community agencies in the Azores.

### Procedures and data collection

This study measured the global QOL, physical and mental health among Azorean deportees. Study participants included a purposeful, convenience sample of twenty five (25) male Azorean migrants **>**18 years of age, who were English or Portuguese speaking, who lived in the US and were deported to the Azores between the years of 2009 and 2013. Study documents (consent form, demographic data sheet and global health tool) were available in both English and Portuguese versions. Participants were able to request which version they preferred, with researchers fluent in each language on site during data collection. A brief overview of the study was provided. Enrolled participants were asked to complete the demographic data sheet which consisted of 13 questions. Following the completion of that form, participants were administered a paper and pencil version of the PROMIS ^®^ Global Health Short Form from the National Institutes of Health (NIH) (2012).

Participants were allowed to ask questions regarding the study and were aware they were free to not participate, or withdraw from the study at any time. By completing the two questionnaires, participants consented and agreed to be part of the research study. Participants were not asked to write their names or any other identifying information on the research documents and a copy of the consent form was distributed to participants for their records at the end of data collection. Data were collected by American and Portuguese nursing students participating in an international community clinical student exchange at two community agencies. Faculty members from the US and Portugal validated the data collected by the students for completeness and accuracy.

### Measures

Demographic items including age, gender, marital status, educational level, place of birth, age at time of immigration to US, number of years spent in the US, and health insurance status were collected using an investigator developed demographic questionnaire. The PROMIS^®^ Global Health tool is a 10 question instrument related to physical and mental health; with participant responses scored into a Global Physical Health component, Global Mental Health component, and EuroQoL (EQ-5D) Index Score, was administered to the study participants. This tool identifies adult participants’ subjective physical and mental health status through self-report. PROMIS^®^ tools can be used across a wide variety of chronic diseases and conditions, and in the general population. The World Health Organization recommends self-report measurements as a globally validated and reliable predictor to identify persons at risk for adverse health outcomes.

The physical and mental health subscales of the PROMIS^®^ Global Short Form were used to collect data on the physical and mental health of Azorean deportees (Hays et al. 2009). Physical and mental health factors were determined through a multi-step process including exploratory and confirmatory factor analysis that demonstrated a two factor solution in 21,133 American adults with chronic illness (Hays et al. 2009). The internal consistency reliabilities of the Physical and Mental Health subscales were 0.81 and 0.86, respectively, by Cronbach’s alpha in previous research (Hays et al. 2009).

The Global Physical Health (GPH) subscale score is determined by summing four items rated on a 5 point Likert type scale (5 excellent to 1 poor). Total scores range from four to twenty, with higher scores indicating better reported physical health. Physical health items include questions about physical health, physical function, pain and fatigue (Hays et al. 2009). Scores on this scale are converted into a T score with 50 representing the average score in the US population. In this study internal consistency reliability was 0.82 by Cronbach’s alpha.

The Global Mental Health subscale (GMH) score is determined by summing the scores on four items also rated on a four point Likert type scale from 5 (excellent) to 1 (poor). Total scores range from four to twenty with higher scores indicating better reported mental health. Mental health items include quality of life, mental health, satisfaction with social activities and emotional problems (Hays et al. 2009). Scores on this scale are converted into a T score with 50 representing the average score in the United States population. Internal consistency reliability in this study by Cronbach’s alpha was 0.63.

Participants also answered two individual questions that were not included in the subscales. The first question was a general rating of overall health from excellent (5) to poor (1). The second question asked participants to rate how well they carry out usual social roles on a scale from excellent (5) to poor (1). EuroQol (EQ-5D) Index Scores were tabulated according to the guidelines presented by Revicki et al. (2009) to determine a global quality of life score. This score measures global quality of life across five dimensions including mobility, self-care, usual activities, pain/discomfort, and anxiety/depression. Scores on the instrument range from −0.109 to 1.0 with higher scores indicating a better reported quality of life (Revicki et al. 2009).

### Statistical analysis

Data were entered into SPSS 22.0 and descriptive statistics were computed on all study variables to determine the presence of random or systematic missing data, significant skewness, and outliers. Descriptive statistics were computed for the each of the single items listed above, as well as for the PH and MH subscales of the PROMIS^®^ Global Short Form and the EQ-5D. Reliability analysis was computed on the PH and MH subscales of the PROMIS^®^ Global Short Form.

## Results

### Sample demographic characteristics

As seen in Table 1, the sample of twenty five men (100 %) were on average middle-aged (M = 48.3 years) with low educational attainment (M = 8.8 years), who spent over three decades (M = 34.4 years) in the US before being deported (M = 3.7 years) to the Azores. A majority (84 %) of the participants were born in São Miguel in the Azores, but left the country and migrated to the US as children (M = 11.7 years of age).

**Table 1:** Demographic characteristics of the sample (n = 25)

Characteristic	n	%	M	SD	Range
Age (in years)			48.3	10.2	28–64
Educational level (in years)			8.8	3.3	2–14
Age at time of migration to US (in years)			11.7	13.3	1–48
Number of years in US			34.4	11.5	10–50
Number of years in Azores since deportation			3.7	1.5	1–7
Global Physical Health Score (4–20)			13.2	4.4	4–20
Global Mental Health Score (4–20)			9.9	3.6	4–16
Quality of Life Score (EQ-5D) (−0.109–1.0)			0.64	0.14	0.33–0.82
Overall health rating (1–5)			2.6	1.4	1–5
Carry out social roles (1–5)			3.2	1.5	1–5
*Gender*					
Male	25	100			
Marital status					
Married	5	20			
Single/widowed	10	40			
Divorced/separated	10	40			
*Current work status*					
Full time	6	24			
Part time	1	4			
Unemployed	13	52			
Retired	1	4			
Disabled	3	12			
*Place of birth*					
São Miguel, Azores	21	84			
Other	4	16			
Health insurance in birth country (no)	15	60			
Current health insurance (no)	12	48			
*Location of migration in US*					
Boston, Massachusetts area	5	20			
Providence, Rhode Island area	5	20			
Southeastern Massachusetts	15	56			

*US* United States

However, more than half (52 %) of the sample were less than seven years of age when migrating to the US Most were unemployed (52 %), not married (80 %) and reported fair QOL (M = 0.64), physical (M = 13.2) and mental (M = 9.9) health. On the single item measures, participants reported fair health overall (M = 2.6) and good ability to participate in social activities (M = 3.2).

### Data from PROMIS^®^ global health tool

When converting physical and mental health scores to T scores as recommended by tool authors, the mean scores of the participants were below the average of 50 found in a prior study of American adults with a variety of chronic illnesses (Hays et al. 2009). The mean physical health score of 13.2 converts to a T score of 42.3 which is close to one standard deviation below the mean of 50 indicating worse physical health (PH) overall in this sample (Hays et al. 2009). The mean mental health score of 9.9 was converted to a T score of 38.8 which is just over one standard deviation below the mean of 50 suggesting that mental health (MH) is more impaired in this population than physical health (PH) (Hays et al. 2009).

### Reported QOL, PH and MH by age of migration and length of time in US

Table 2 displays mean scores of reported quality of life, physical and mental health of deportees who moved to the US as children (age ≤17 years) and as adults (age >18 years) computed with descriptive statistics. Similar statistics were computed for the same variables in those who had been in the US for less than 25 years and greater than or equal to 25 years. QOL (0.652 vs. 0.609) and PH (13.9 vs. 11.8) mean scores were higher in those who migrated to the U.S. at a younger age (≤17 years); whereas MH (9.4 vs. 11.0) was lower in the same age group. QOL (0.615 vs. 0.644) and PH (12.6 vs. 13.4) mean scores were lower in those who had been in the US for less than 25 years; while MH (11.0 vs. 9.7) mean scores were lower in those who had been in the US for greater than or equal to 25 years, respectively.

**Table 2:** Reported QOL, PH and MH by age of migration and length of time in US

Characteristic	Age at migration to US		Length of time in US	
	≤17 years	>17 years	<25 years	≥25 years
	M	M	M	M
Quality of life	0.652	0.609	0.615	0.644
Physical health	13.9	11.8	12.6	13.4
Mental health	9.4	11.0	11.0	9.7

*US* United States

### Reported QOL, PH and MH by length of time since deportation and marital status

Mean scores of reported QOL, PH and MH of men who were deported ≤3 years versus >3 years were computed using descriptive statistics. With similar statistics the same variables were computed in married versus unmarried persons (widowed/divorced/single). The results are reported in Table 3. QOL (0.632 vs. 0.637) had lower mean scores in those who were deported more recently (≤3 years), whereas PH (13.3 vs. 12.8) and MH (10.4 vs. 9.6, *p* = 0.63) mean scores were higher in those deported greater than or equal to 3 years. QOL (0.635 vs. 0.671), PH (13.2 vs. 14.0) and MH (9.1 vs. 10.2) mean scores were lower in those who were married versus those who were unmarried.

**Table 3:** Reported QOL, PH and MH by length of time since deportation and marital status

Characteristic	Length of time deported		Marital status	
	≤3 years	>3 years	Married	Unmarried
	M	M	M	M
Quality of life	0.632	0.637	0.635	0.671
Physical health	13.3	12.8	13.2	14.0
Mental health	10.4	9.6	9.1	10.2

*US* United States

## Limitations

Limitations of this study included having a small sample (N = 25) from a relatively homogenous group of male deportees whose focus was on a similar pattern of migration and deportation between the US and the Azores. This small sample may have an effect on the internal consistency reliability of the global mental health subscale (Cronbach’s alpha 0.63). It would be difficult at this juncture to establish external validity due to sample size; however, sufficient details of the results of this study are provided to allow the reader to evaluate the applicability of this data to other contexts. Furthermore, all participants were recruited from two community agencies committed to providing aid and support for deportees toward reintegration; leaving the QOL, PH and MH of deportees who choose not to utilize available support services unknown.

## Discussion

The PROMIS^®^ Global Health Short Form was used to measure the self-reported QOL, PH and MH of 25 men deported from the US to the Azores between the years 2009–2013. Overall, results showed that participants Global Physical Health and Global Mental Health subscale scores were 13.2 and 9.9 respectively, on a scale that ranged from 4 to 20 (higher scores indicate better reported health) (Hays et al. 2009); suggesting that overall PH and MH in these men were lower than the mean across all study scores, with their MH being more impaired than PH in this population.

In addition, the overall health rating from excellent (5) to poor (1) was 2.6; and participants’ ability to carry out usual social activities with an overall health rating from excellent (5) to poor (1) was 3.2. And finally, the EQ-5D Index score with an instrument range of -0.109-1.0 with higher scores indicating better reported QOL was reported as 0.64 in this population.

Study findings also identified higher mean MH scores (11.0 vs. 9.4) in those >18 years versus ≤17 years of age at time of migration to the US, and higher QOL (0.644 vs. 0.615) and PH (13.4 vs. 12.6) mean scores among those who were in the US ≥25 years and <25 years. Next, QOL mean scores (0.637 vs. 0.632) were higher in those who were deported >3 years and ≤3 years respectively. Finally, those who were unmarried reported higher QOL, PH and MH mean scores (0.671 vs. 0.635; 14.0 vs. 13.2; and 10.2 vs. 9.1) respectively.

Although it is difficult to make generalizations due to small sample and use of descriptive statistics, it is likely that other variables, such as: (a) Reintegration post deportation, (b) support; and, (c) and family structure are contributing to low self-reported scores that require further investigation. It is also plausible that this group could have been considered vulnerable following initial migration to the US, as immigrants often face obstacles to health care access, language and cultural barriers, economic and social difficulties, and fear of deportation (Fleishman et al. 2015; Hacker et al. 2012). It has been noted that approximately 25 % of deportees receive social support services prior to deportation proceedings (Nunes Rocha and Borralho 2012). Participants in this study described a low level of education (M = 8.8 years), and the majority (52 %) were unemployed. The process of deportation might lend itself to an exacerbation of their vulnerability and negatively impact their PH and MH, although measuring PH and MH scores pre deportation and post deportation may elucidate differences in self-perceived health following this event.

There are few studies in the literature pertaining to nursing research conducted with this vulnerable group and the effect of deportation on the health and well-being of individuals, families and communities. Understanding this perspective is essential to conduct future intervention research that supports family transition and related health implications. More research is needed to explore self-reported health in a larger heterogeneous sample of deportees from different spans of time, geographical perspective, and those utilizing no support services, or varying degrees of support and among diverse family unit structures.

## Conclusions

Based on the findings of this study, the PROMIS^®^ Global Health Short Form was useful in measuring self-reported QOL, physical and mental health of deportees from the US to the Azores. Study results suggest the enrolled deportees were less healthy than the general population. In spite of the lack of significant findings, overall scores were lower than mean tool scores indicating a need for more research in this vulnerable group related to support, clinical practice and health policy to improve their overall QOL and health through future intervention work. Future research and tailored initiatives regarding the overall health of deportees, with a focus on quality of life and mental health should be conducted to better understand their impact on reintegration.

## References

[CR1] Ayon DR (2009) Developing the US-Mexico border region for a prosperous and secure relationship: the impact of Mexican migration on border proximity on local communities. http://bakerinstitute.org/research/the-impact-of-mexican-migration-and-border-proximity-on-local-communities/. Accessed 19 Jan 2016

[CR3] Bernstein N (2004) A mother deported, and a child left behind. New York Times. Boston College. (2014). Center for Human Rights and International Justice. http://www.bc.edu/centers/humanrights/projects/deportation/aboutpdhrp.html. Accessed 11 Dec 2015

[CR4] Blake G (2014). America’s deadly export: evidence from cross-country panel data of deportation and homicide rights. Int Rev Law Econ.

[CR5] Brabeck K, Brintin Lykes M, Hunter C (2014). The psychological impact of detention and deportation on US migrant children and families. Am J Orthopsychiatry.

[CR6] Department of Homeland Security (2013) Immigration statistics. http://www.dhs.gov/immigration-statistics. Accessed 3 Nov 2015

[CR7] Dolan C, Schuster L, Merefield M (2016) The impact of deportation: some reflection on current practice. http://www.refugeelawproject.org/files/briefing_papers/The_Impact_of_Deportation.pdf. Accessed 3 March 2016

[CR8] Fleishman Y, Willen S, Davidovitch N, Mor Z (2015). Migration as a social determinant of health for irregular migrants: Israel as case study. Soc Sci Med.

[CR9] Gushulak B, MacPherson D (2006). The basic principles of migration health: population mobility and gaps in disease prevalence. Emerg Themes Epidemiol.

[CR10] Hacker K, Chu J, Arsenault L, Marlin R (2012). Providers perspectives on the impact of immigration and customs enforcement (ICE) activity on immigrant health. J Health Care Poor Underserved.

[CR11] Hays RD, Bjorner JB, Revicki DA, Spritzer KL, Cella D (2009). Development of physical and mental health summary scores from the patient-reported outcomes measurement information system (PROMIS) global items. Qual Life Res.

[CR12] Hilfinger Messias D, McEwen M, Clark L (2015). The impact and implications of immigration on individual and collective health in the United States. Nurs Outlook.

[CR13] Human Rights Watch (2009) Forced apart (By the numbers): Non-citizens deported mostly for non-violent offenses. https://www.hrw.org/report/2009/04/15/forced-apart-numbers/non-citizens-deported-mostly-nonviolent-offenses. Accessed 4 Nov 2015

[CR14] Immigration in America (2012) Illegal immigration reform and immigrant responsibility act of 1996. http://immigrationinamerica.org/577-illegal-immigration-reform-and-immigrant-responsibility-act-of-1996.html. Accessed 1 Nov 2015

[CR15] International Organization for Migration (2013) World migration report. http://www.iom.int/files/live/sites/iom/files/What-WeDo/wmr2013/en/WMR2013_Overview_EN_final.pdf. Accessed 1 March 2016

[CR16] Library of Congress (2010) The Portuguese in the United States. https://www.loc.gov/rr/hispanic/portam/. Accessed 7 Feb 2016

[CR17] Lonegan B (2007). American diaspora: the deportation of lawful residents from the United States and the destruction of their families. NY Univ Rev Law Soc Change.

[CR18] Moniz M (2004) Exiled home: criminal deportee forced return migrants and transnational identity: the Azorean example. Dissertation

[CR19] Morris JE, Palazuelos D (2015). The health implications of deportation. J Health Care Poor Underserved.

[CR20] National Institutes of Health (2012) PROMIS ^®^ Global Health Short Form. http://www.nihpromis.org/. Accessed 3 Apr 2015

[CR21] Nunes Rocha GP, Borralho A (2012) Emigrantes deportados nos acores. Universidade Dos Acores

[CR22] Revicki DA, Kawata AK, Harnum N, Chen W-H, Hays RD, Cella D (2009). Predicting EuroQol (EQ-5D) scores from patient-reported outcomes measurement information system (PROMIS) global items and domain item banks in a United States sample. Qual Life Res.

[CR23] Richardson GV (1971) 403 US

[CR24] Rosenblum M, Meissner D (2014) Migration Policy Institute. The deportation dilemma: Reconciling tough and humane enforcement, p 71

[CR25] Shaughnessy HV (1952) 342 US

[CR26] Suarez-Orozco C, Yoshikawa H, Teranishi R, Suarez-Orozco M (2011). Growing up in the shadows: the development implications of unauthorized status. Harv Educ Rev.

[CR27] Vallet M (2012). Post deportation health: a humanitarian assessment. http://forms.nomoredeaths.org/wp-content/uploads/2014/10/DIS_Report-NMD-Dec2012final.pdf. Accessed 12 Dec 2015

[CR28] Williams JR (2007). In pursuit of their dreams: a history of Azorean immigration to the United States.

[CR29] World Health Organization (1946) Preamble to the Constitution of the World Health Organization as adopted by the International Health Conference, New York, 19–22 June, 1946; signed on 22 July 1946 by the representatives of 61 States (Official Records of the World Health Organization, No. 2, p. 100) and entered into force on 7 April 1948. Accessed 4 Nov 2016

[CR30] World Health Organization (2015) Migrant Health. http://www.who.int/hac/techguidance/health_of_migrants/en/. Accessed 3 April 2015

